# Expression Profile of the *SCNN1G* Gene and Its Association of Polymorphisms With Eggshell Quality Traits in the Potchefstroom Koekoek Chicken Breed

**DOI:** 10.1155/vmi/5917044

**Published:** 2026-06-17

**Authors:** Kagisho Madikadike Molabe, Thobela Louis Tyasi, Vusi Mbazima

**Affiliations:** ^1^ School of Agricultural and Environmental Sciences, Department of Agricultural Economics and Animal Production, University of Limpopo, Private Bag X1106, Sovenga, 0727, Limpopo, South Africa, ul.ac.za; ^2^ School of Molecular and Life Sciences, Department of Biochemistry Microbiology and Biotechnology, Private Bag X1106, Sovenga, 0727, Limpopo, South Africa

**Keywords:** eggshell thickness, marker–trait association, mRNA expression, single-nucleotide polymorphism

## Abstract

Enhancement of the production of eggs with good‐quality eggshells is of serious interest to ensure food security as eggs are assessed mostly by their eggshell quality. A high rate of cracked eggs has been reported to cause economic loss, increasing the risk of bacterial infections leading to low egg production. Hence, the study was conducted to determine the genetic variation of the *Sodium Channel Epithelial 1 Subunit Gamma* (*SCNN1G*) gene in improving eggshell thickness of the Potchefstroom Koekoek chicken breed. The study was conducted at the University of Limpopo with 100 Potchefstroom Koekoek point‐of‐lay chickens raised intensively. A total of 350 eggs were randomly collected at the 31^st^ to 39^th^ weeks for the collection of internal, external, and eggshell quality traits, and tissues (shell gland, magnum, and isthmus) and blood samples were collected for molecular analysis. Statistical Analysis System Version 9.4 (SAS, 2020) software was used for data analysis. DNA sequencing, general linear model (GLM), and quantitative reverse transcription PCR (RT‐qPCR) were used to achieve the objective. DNA sequence analysis revealed two SNPs (A > T at position 2587 and C > T at position 1013 in the coding region). Marker–trait association showed shell thickness (ST) having a significant association (*P* < 0.05) with all the genotypes where genotype AA was associated with thicker ST than genotype AT; then, genotype CC was associated with thicker ST than genotype CT. The RT‐qPCR results demonstrated significant difference (*P* < 0.05) in *SCNN1G* mRNA expression levels where the highest expression level was observed in magnum, followed by isthmus and shell gland, respectively. The findings of the study could give evidence on the relevance of genetic markers, mRNA expression analysis, and other molecular biology tools that could be used during genetic selection programs aimed at improving egg production traits specifically eggshell quality.

## 1. Introduction

Enhancement of the production of eggs with good‐quality eggshells is of serious interest to ensure food security; moreover, eggs are assessed mostly by their eggshell quality [1]. Egg production displayed an imperative increase of 30% in Nigeria and South Africa from 2013, as reported by [2]. Globally, about 1.3 trillion eggs are laid per year by about 7 billion chickens [3]. Eggs are an essential component to the human diet, playing a role as a source of animal protein, fatty acids, vitamins, and minerals [4]. Potchefstroom Koekoek is a synthetic indigenous breed layer developed in South Africa which can produce eggs with brown shells having an average weight of 55.7 g [5]; additionally, its strong bone formation enables its movements and strong ability to run and fly, using this as a protective mechanism against danger and predators [6]. As a dual‐purpose breed, cocks and culled hens are used for meat production, and their meat, due to its tenderness and nutritional content, is preferred over the commercial broiler hybrids [4]. The quality of the egg is determined by both internal and external characteristics [7]. External characteristics, particularly eggshell traits, are the major focus of this study. Eggshell is the outer part of the egg which is essential as it protects the content of the egg from physical damage and microbial contamination [8] as well as enables the growth and development of the embryo [9].

A high rate of cracked and broken eggs has been reported to cause economic loss, increasing the risk of bacterial infections, which in turn lowers egg production [7]. Deteriorated eggs negatively affect the enterprises in the egg production industry [8]. As documented by [10], eggshell quality is affected by many factors, including disease, nutrition, and environment. In addition, the production of eggs also involves biochemical processes and gene expression [1]. Gene‐associated markers and mRNA expression analysis gained approval in animal husbandry across many countries [11]. The increased use of DNA‐based markers plays a role in improving economically important traits and increasing production in terms of both composition and quality to support food security [12].

With technology development, molecular genetic techniques, such as single‐nucleotide polymorphisms (SNPs) and mRNA expression analysis, have gained acceptance in breeding of livestock, such as cattle, sheep, goats, and chickens due to their potential to assist in genetic selection for future production chickens [13]. According to [14], genetic markers, such as candidate genes, are broadly utilized in national breeding programs to improve production. Previous investigation by [7] showed numerous polymorphisms in different chicken genes that have an impact on egg quality traits [15]. Genetic diversity of *parathyroid hormone* gene and its association with eggshell quality were investigated, and synonymous SNP (A2205G) was identified, which increases the translation and mRNA stability in chicken. Furthermore, eggshell breakage and eggshell percentage were affected by genotypes GG and AG than genotype AA [7]. Another promising candidate gene for improving eggshell quality is the Sodium Channel Epithelial 1 Subunit Gamma (SCNN1G) gene. This gene has been reported to play a role in calcium homeostasis by regulating the levels and liberation of calcium from bones and kidneys [16], and it is also involved in transmembrane transport of calcium during eggshell formation, also affecting the eggshell quality, especially eggshell strength and eggshell thickness.

Based on our knowledge, there is limited literature on the genetic analysis of the *SCNN1G* gene and its association with eggshell thickness of Potchefstroom Koekoek. Therefore, the findings of the current study may assist breeders in advising egg producers on strategies to improve the eggshell quality of their chickens. In addition, the study could also give evidence on the relevance of genetic markers, mRNA expression analysis, and other molecular biology tools that could be used during genetic selection programs aimed at improving egg production traits specifically eggshell quality.

## 2. Materials and Methods

### 2.1. Study Site

The current study was conducted at the University of Limpopo experimental farm located 10 km north–west of the main campus and lies at latitude of 27.55° South and longitude of 24.77° East; during summer, the farm receives annual rainfall of < 400 mm, with ambient temperatures of > 30°C and > 25°C, during winter [17].

### 2.2. Animal Population, Management, and Research Design

The study used 100 Potchefstroom Koekoek point‐of‐lay chicken, which were purchased from Angel Feeds in Polokwane, as experimental animals. The breed was developed in South Africa during the 1950s by Marais at Potchefstroom Agricultural College; the name explains its barred color pattern, as it results from the crossbreeding of White Leghorn, Black Australorp, and barred Plymouth. Potchefstroom koekoek’s meat and eggs are significant and serve as a source of nutrients, generating income for rural areas and playing a role in poverty alleviation, with an impact on the country’s economy; hence, they are of interest to be involved in breeding programs. They were kept and raised under an intensive management system where they were kept in a medium‐tier cage system with 3 hens in each cage (30 × 30 × 25.5 cm). Feed and water were given at all times. They were provided with layers’ mash containing 16% crude protein and 11.97 MJkg/DM throughout the laying period, and standard normal management protocol was followed.

### 2.3. Data Collection

#### 2.3.1. Phenotypic Measurements

A total of 350 eggs were randomly collected from the chickens on the 31^st^ to 39^th^ weeks. Traits recorded (measured three times, and the average was considered) as external egg quality traits were egg weight (g), egg length (mm), egg width (mm), egg volume (cm^3^), and egg shape index (%). Egg length and width measurements were collected using a vernier caliper. Balanced weighing scale (crate scale 6 kg × 01 kg) of Tronic Services, South Africa, was used to weigh the eggs. Equations as suggested by [18] were used to calculate egg shape index and egg volume.

Eggshell quality traits, such as eggshell thickness (mm), shell ratio (%), shell weight (g), shell strength (N), shell surface area (cm^3^), and unit surface shell weight (g/cm^2^), were also collected. Micrometer gauge (QCT shell thickness [ST] micrometer, TSS, England) was used to determine the thickness of the egg after 72 h of exposure, while the QCT shell strength tester was employed to determine the strength. The electronic scale weighed the eggshell, and the equation by [19] determined the shell ratio.

#### 2.3.2. Collection of Tissues (Shell Gland, Magnum, and Isthmus)

A total of five (5) chickens were randomly selected for slaughter at 46 weeks. The chickens were slaughtered without severing the head, but by cutting through the esophagus, carotid arteries, the throat, trachea, and jugular veins. The chickens were then dissected, and the shell gland, magnum, and isthmus were collected and rinsed with ice‐cold isotonic saline. Aluminum foil was used to cover about 500 mg of sample tissue of the shell gland, magnum, and isthmus and placed at −20°C and frozen in liquid nitrogen until use.

#### 2.3.3. Blood Collection and Amplification of *SCNN1G*

A total of 100 blood samples (2‒3 mL each) were collected at 46 weeks of age with the help of a veterinarian, using 21‐gauge needles and 5‐mL syringes on a one‐off interval. The blood samples were kept in 10 mL ethylenediaminetetraacetic acid (EDTA) tubes and kept at 4°C till use. DNA was extracted from the blood samples using Norgen’s Genomic DNA Isolation Kit (Norgen Biotek Corp., Canada). A NanoDrop spectrophotometer (Thermo Scientific) was employed to assess the purity and concentration of DNA samples. The *SCNN1G* gene was amplified by polymerase chain reaction (PCR) with primers (Table 1) designed using CLC Main Workbench 5 and Primer Premier 6.1 software, as described by [9]. The PCR was carried in 25 μL mixture containing 100 ng genomic DNA, 10x PCR buffer, 0.5 μL of each primer (5 pmol), and 12.5 μL of Farazist Avaran Sorengostar master mix and deionized water. The PCR cycling conditions were conducted in a thermocycler as follows: an initial denaturation step at 940°C for 10 min followed by 35 cycles of denaturation at 940°C for 30 s, annealing at 550°C for 30 s, extension at 720°C for 45 s, and a final extension at 720°C for 10 min.

**TABLE 1:** Primers used to amplify the *SCNN1G* gene.

Primer name	Sequence (5′⟶3′)	Tm °C	Product size
*SCNN1G*‐F	GCGGGATATGCCATTCATTACTGC	61	589
*SCNN1G*‐R	GCTCCGTGTCGGGATAGAAG		

#### 2.3.4. DNA Sequencing

The *SCNN1G* gene segment amplified by PCR was sent to Inqaba Biotechnology in Pretoria, South Africa, for DNA sequencing, and the NCBI/BLAST/blastin site was used to perform sequence alignment.

#### 2.3.5. mRNA Expression Analysis of the *SCNN1G* Gene

Samples (shell gland, magnum, and isthmus tissues) were finely minced, and RNA was extracted using the Quick RNA Miniprep Plus Kit (R1058) following the manufacturer’s protocol. Quality and concentration of RNA samples were measured using the NanoDrop One Microvolume UV‐Vis Spectrophotometer (Thermo Fisher Scientific). First‐strand cDNA was synthesized using LunaScript RT Super Mix Kit (New England Biolabs, Ipswich, MA, USA) according to the manufacturer’s instructions in a total volume of 20 μl containing 1 ug total RNA. The cDNA program comprised an initial cycle (primer annealing) at 25°C for 2 min, followed by cDNA synthesis cycle at 55°C for 10 min and lastly heat inactivation at 95°C for a minute.

Quantitative RT‐PCR was then performed in 96‐well plates with the Luna Universal qPCR Master Mix (1X) (New England Biolabs, Ipswich, MA, USA) using dye‐based qPCR assay. Each reaction contained 1 ul of cDNA template, 0.25 μM forward (5′‐TGGGTATGGAGTCCTGTGGT‐3′) and reverse (5′‐AGGGCTGTGATCTCCTTCTG‐3′) primers, and 1X Luna Universal qPCR Master Mix. The qPCR program started by an initial denaturation cycle at 95°C for 60 s followed by denaturation cycle at 95°C for 15 sec, annealing at 25°C for 2 min, and extension at 60°C for 30 s for 30 cycles.

The reactions were run on CFX96 Real‐Time PCR System (Bio‐Rad) following a standard two‐step PCR program as suggested by Luna Universal qPCR Master Mix manual. Three technical replicates were run for each cDNA sample. Amplification of different input templates was evaluated based on the quantification cycle (Cq) value. The beta‐actin (ACTB) gene was used as an internal control (housekeeping) gene. The method by [20] was used to quantify the relative gene expression levels.

### 2.4. Ethical Approval

The current study was approved by the University of Limpopo Animal Research and Ethics Committee (ULAREC) with project number: AREC/55/2023: PG before the commencement of the study.

### 2.5. Statistical Analysis

Data were analyzed at the 5% significance level using Statistical Analysis System Version 9.4 (SAS, 2020) software. DNA sequencing, general linear model (GLM), and quantitative reverse transcription PCR (RT‐qPCR) were used to achieve the objective. The following GLM was used for marker–trait association analysis:

(1) Yij=u+Gi+eij

where

*Y*_*i* *j*_ = phenotypic values of *i*th trait on *j*th genotype,

*μ* = population mean, *G*_*i*_ = fixed effect of *i*th genotype, and

*e*_*i* *j*_ = random residual error.

## 3. Results

### 3.1. PCR Amplification and Sequencing of the *SCNN1G* Gene

#### 3.1.1. PCR Analysis

**FIGURE 1:**
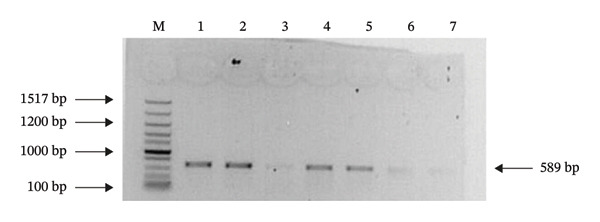
PCR products of the *SCNN1G* gene from the Potchefstroom Koekoek chicken breed. M, DL 1517 DNA marker (100 bp, 600 bp, 900 bp, and 1000 bp, respectively).

The *SCNN1G* gene from the Potchefstroom Koekoek chicken breed was amplified using PCR and resulted in an amplicon size of 589 bp, which corresponds to the expected gene fragment size (Figure 1).

#### 3.1.2. *SCNN1* Gene Sequence

**FIGURE 2:**
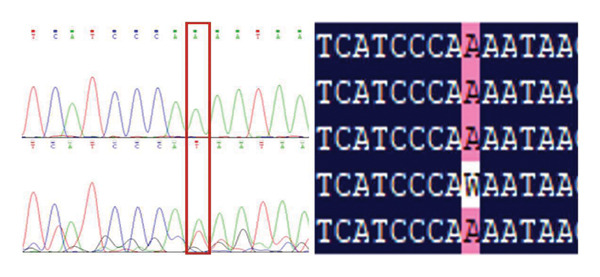
Nucleotide sequence analysis showing A2587T transversion of the SCNN1G gene in Potchefstroom Koekoek chicken.

**FIGURE 3:**
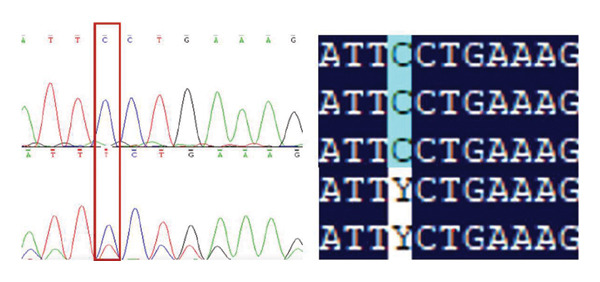
Nucleotide sequence analysis showing C1013T transition of the *SCNN1G* gene in Potchefstroom Koekoek chicken.

Sequencing analysis of the *SCNN1G* gene was performed using DNAMAN and Chromatogram software to identify SNPs. The DNA sequence analysis of the *SCNN1G* (accession no: XM_015294500.3) revealed two SNPs: an A > T at position 2587 (Figure 2) and C > T at position 1013 in the coding region (Figure 3). The polymorphism located at position 2587 showed a transversion from adenine (A) to thymine (T) implicating a nonsynonymous amino acid exchange from lysine to isoleucine. On the other side, the cytosine (C) to thymine (T) transition at position 1013 showed a synonymous amino acid exchange, resulting in no change in the amino sequence.

#### 3.1.3. Gene Pairwise Alignment

**FIGURE 4:**
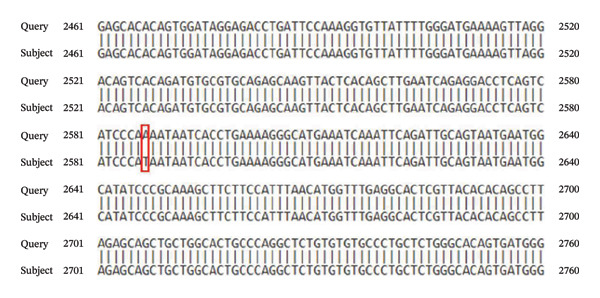
Gene pairwise alignment on position 2587. The red color shows the SNP position.

**FIGURE 5:**
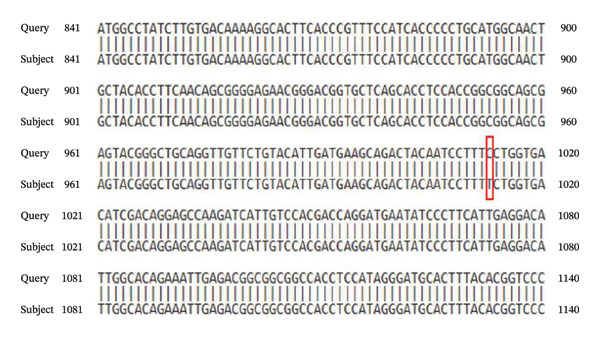
Gene pairwise alignment at position 1013. Highlight in red color shows the SNP position.

The sequences were blasted for DNA sequence pairwise alignment using NCBI sequence alignment. The pairwise alignment results of *SCNN1G* gene sequence demonstrated the location of *SCNN1G* gene SNP (A > T) (Figure 4) and the location of C > T (Figure 5).

#### 3.1.4. Genotype and Allelic Frequencies

Genotypic and allelic frequencies for *SCNN1G* SNP (Table 2) were calculated using population genetic analysis. The results showed that two alleles (A and T) and two genotypes (AA and AT) were revealed at the A > T SNP, with allele A having a higher frequency than of allele T, and the AT genotype showing lower frequency than genotype AA. The C > T SNP resulted in two genotypes (CC and CT) and two alleles (C and T), with the CC genotype showing a higher frequency than the CT genotype. Additionally, allele C showed higher frequency than allele T. The genetic equilibrium of the population, based on the Hardy–Weinberg theorem, was measured using the chi‐square (*χ* ^2^) test, which showed that the allelic and genotypic frequencies of both populations are under Hardy–Weinberg equilibrium.

**TABLE 2:** Genotype and allelic frequencies of *SCNN1G*.

SNP	Genotype	Number of animals	Allele	Allelic frequencies	Genotypic frequencies	*χ* ^2^	*P* value
A > T	AA	73	A	0.87	0.73	2.44	0.02
	AT	27	T	0.14	0.27		
C > T	CC	81	C	0.91	0.81	0.57	
	CT	19	T	0.10	0.19		

#### 3.1.5. Polymorphism Information Analysis

The population genetic analysis was used to determine the genetic diversity and polymorphism information analysis. The genetic diversity parameters, heterozygosity (Ho), expected heterozygosity (He), effective allele number (Ne), and polymorphism information content (PIC) for the *SCNN1G* gene are presented in Table 3. Gene homozygosity was greater than gene heterozygosity in both populations, with effective allele numbers of 1.30 (A > T) and 1.22 (C > T) SNP. These findings suggest a moderate‐level polymorphism.

**TABLE 3:** *SCNN1G*’s genetic diversity.

SNP	Gene heterozygosity (H_*e*_)	Gene homozygosity (Ho)	Effective allele number (Ne)	Polymorphism information content (PIC)
A > T	0.27	0.77	1.30	0.21
C > T	0.19	0.83	1.22	0.16

#### 3.1.6. Association Between the SNP of the *SCNN1G* Gene and Eggshell Quality Traits

The associations between SNPs and the investigated traits are presented in Tables 4 and 5 generated through GLM. Among the traits investigated, only ST showed a significant association (*P* < 0.05) with all the genotypes. The results indicated that genotype AA was remarkably related to thicker ST than genotype AT (Table 4). The genotype CC was associated with thicker ST than genotype CT (Table 5).

**TABLE 4:** Association between A2587T polymorphism of *SCNN1G* gene and eggshell quality traits.

Traits	Genotype		*P* value
	AA	AT	
	(mean ± SE)		
SW (g)	5.94 ± 0.09	5.10 ± 0.14	0.75
SR (%)	34.39 ± 0.61	36.27 ± 1.05	0.11
SSA (cm^3^)	74.12 ± 0.63	74.82 ± 0.10	0.56
USSW (g/cm^2^)	0.08 ± 0.00	0.08 ± 0.00	0.70
ST (mm)	0.66 ± 0.04^a^	0.26 ± 0.02^b^	< 0.00
SS (N)	4.74 ± 0.21	4.27 ± 0.31	0.23

Abbreviations: SR = shell ratio, SS = shell strength, SSA = shell surface area, ST = shell thickness, SW = shell weight, and USSW = unit shell surface.

^a,b^Different superscript on the same row shows the significant difference (*p* < 0.05).

**TABLE 5:** Association between C1013T polymorphism of *SCNN1G* and eggshell quality traits.

Traits	Genotype		*P* value
	CC	CT	
	(mean ± SE)		
SW (g)	5.95 ± 0.89	6.00 ± 0.16	0.78
SR (%)	34.55 ± 0.58	36.12 ± 1.32	0.25
SSA (cm^3^)	74.26 ± 0.58	75.03 ± 1.25	0.57
USSW (g/cm^2^)	0.08 ± 0.00	0.08 ± 0.00	0.87
ST (mm)	0.61 ± 0.04^a^	0.30 ± 0.04^b^	< 0.00
SS (N)	4.67 ± 0.18	4.64 ± 0.17	0.74

Abbreviations: SR = shell ratio, SS = shell strength, SSA = shell surface area, ST = shell thickness, SW = shell weight, and USSW = unit shell surface.

^a,b^Different superscript on the same row shows the significant difference (*p* < 0.05).

#### 3.1.7. Gene Expression

**FIGURE 6:**
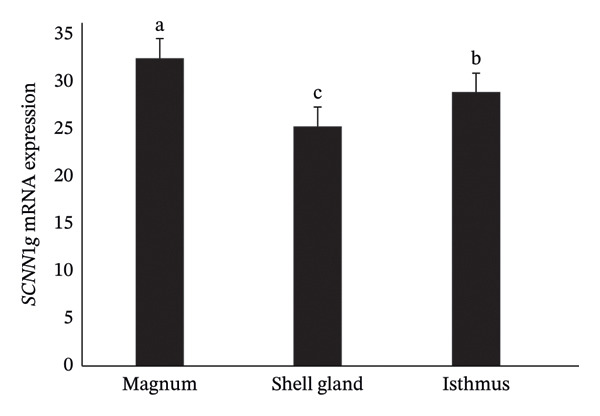
*SCNN1G* mRNA expression level in the reproductive organs (magnum, sheep gland, and isthmus) of the Potchefstroom Koekoek chicken breed.

The RT‐qPCR was employed to determine the expression level of the *SCNN1G* gene in the reproductive organs (magnum, isthmus, and shell gland) of chicken breed (Figure 6). The findings showed a significant difference (*P* < 0.05) in *SCNN1G* mRNA expression levels in the organs of interest. The highest expression level was observed in magnum, followed by isthmus, while the shell gland showed the lowest expression of *SCNN1G* mRNA.

## 4. Discussion

As documented by [3], eggs with poor eggshell quality decrease production and also the possibility of being selected by the user due to the damaged presentation. Moreover, the defect of eggshell might also be due to genetic predisposition [15]. Hence, the current study was conducted to investigate eggshell thickness quality in the Potchefstroom Koekoek chicken breed of South Africa, with the aim of providing evidence on genetic diversity that could be used in genetic selection to improve egg production traits, specifically eggshell quality.

Sequence analysis of the *SCNN1G* gene revealed two SNPs at different locations. Specifically, an A > T and C > T were recognized at positions 2587 and 1013 of the coding region, respectively. These SNPs were classified as nonsynonymous and synonymous, respectively. Further findings on gene–marker association showed that ST was noted to be the only trait associated with the observed genotypes. Chickens with genotypes AA and CC were associated with thicker ST, while genotypes AT and CT were associated with thinner ST. Furthermore, gene expression results in significant difference in *SCNN1G* mRNA expression levels in magnum, isthmus, and shell gland, with the highest expression observed in magnum, followed by the isthmus and the lowest in the shell gland.

Studies investigating genes associated with improving eggshell thickness are very limited [9]. Study conducted by [21] on the sodium channel gene family found that it is specifically expressed in the hen uterus and is associated with eggshell quality traits; additionally, they identified the rs15009191 SNP with three genotypes, where genotypes CC and TC were associated with thicker estimated ST, while genotype TT was significantly associated with thinner estimated ST. In contrast, findings by [9] disagree with the current study, reporting no association between genotypes and eggshell strength. Furthermore, the study reported a high expression level of the *SCNN1G* gene in the uterus compared to the duodenum and magnum, implying the gene’s involvement in Na+ absorption by the uterine glandular cells in the apical membrane. The observed discrepancies may be attributed to the use of different breeds.

According to [21], sodium channels can affect eggshell quality, especially eggshell strength and eggshell thickness. Polymorphism in genes associated with eggshell organic matrix was considered to be related to eggshell breaking strength, thickness, and dynamics [22]. In this study, the *SCNN1G* gene showed high expression levels in the magnum and was significantly associated with eggshell traits in chickens. These findings provide evidence that genetic variation in *SCNN1G* influences eggshell quality.

## 5. Conclusion

The study concludes that the identified genetic markers (A2587T and C1013T) are significantly associated with eggshell quality trait, specifically eggshell thickness, and are strongly linked to thicker. This implies that they may be used as the genetic markers for the improvement of economically important traits. Additionally, the mRNA expression levels of the *SCNN1G* gene may serve as a valuable indicator in selection and breeding programs for genetic improvement of eggshell thickness of the Potchefstroom Koekoek chicken breed. However, more studies need to be conducted to explore the genetic variation and gene expression profiles using different breeds and larger sample sizes.

## Funding

The authors would like to thank the National Research Foundation (NRF), reference number (PMDS22051410688), for its financial support.

## Conflicts of Interest

The authors declare no conflicts of interest.

## Data Availability

Data are available through the corresponding author.
